# Sensitivity and breadth of detection of high-throughput sequencing for adventitious virus detection

**DOI:** 10.1038/s41541-020-0207-4

**Published:** 2020-07-17

**Authors:** Robert L. Charlebois, Sarmitha Sathiamoorthy, Carine Logvinoff, Lucy Gisonni-Lex, Laurent Mallet, Siemon H. S. Ng

**Affiliations:** 1 Analytical Sciences, Sanofi Pasteur, Toronto, ON M2R 3T4 Canada; 2 Turnstone Biologics, Ottawa, ON K1S 3V5 Canada; 3 Analytical Sciences, Sanofi Pasteur, Marcy L’Étoile, France

**Keywords:** Immunology, Medical research

## Abstract

High-throughput sequencing (HTS) is capable of broad virus detection encompassing both known and unknown adventitious viruses in a variety of sample matrices. We describe the development of a general-purpose HTS-based method for the detection of adventitious viruses. Performance was evaluated using 16 viruses equivalent to well-characterized National Institutes of Health (NIH) virus stocks and another six viruses of interest. A viral vaccine crude harvest and a cell substrate matrix were spiked with 22 viruses. Specificity was demonstrated for all 22 viruses at the species level. Our method was capable of detecting and identifying adventitious viruses spiked at 10^4^ genome copies per milliliter in a viral vaccine crude harvest and 0.01 viral genome copies spiked per cell in a cell substrate matrix. Moreover, 9 of the 11 NIH model viruses with published in vivo data were detected by HTS with an equivalent or better sensitivity (in a viral vaccine crude harvest). Our general-purpose HTS method is unbiased and highly sensitive for the detection of adventitious viruses, and has a large breadth of detection, which may obviate the need to perform in vivo testing.

## Introduction

The potential contamination of biopharmaceuticals with adventitious (unintentionally introduced) viruses poses a serious safety risk and threatens public confidence in the use of biopharmaceuticals. This is particularly true in the case of vaccines administered to large numbers of healthy people, including children^1,2^. There have been a number of instances over previous decades where evidence for the presence of adventitious virus contamination in a marketed vaccine product has threatened public trust in immunization programs^3^. Examples include the detection of simian virus 40 (SV40) in the early polio vaccine in the 1960s, the finding of reverse transcriptase in measles and mumps vaccines in 1995 and of porcine circovirus (PCV) nucleic acid sequences and/or infectious circovirus in rotavirus vaccines in 2010^3,4^.

These viruses could be unintentionally introduced at various manufacturing stages and may originate from multiple sources including raw materials, the cell substrate or the environment. Implementation of Good Manufacturing Practise and close monitoring of the manufacturing process can help reduce the likelihood of viral contamination. Therefore, adventitious virus testing at various stages of the manufacturing process is an integral part of the safety assessment for vaccines and other biological products, and there are well-defined regulatory requirements to ensure that these products are absent of adventitious viruses^5,6^.

Gaps exist in the current compendial adventitious virus testing package where some viral families are not detected or incompletely detected by the compendial methods^7–9^. Non-specific adventitious virus testing of biological materials has typically included in vivo tests and cell culture-based in vitro tests, the breadth and sensitivity of which are presumed from historical experience rather than from systematic assay validation^9^. In addition, routine testing for adventitious viruses following the compendial requirements generally requires testing the cell substrate and the viral crude harvest (at seed lot and/or bulk levels). Both of these manufacturing stages present complex matrices for in vivo and in vitro testing. Targeted PCR-based virus detection methods require prior knowledge of the adventitious virus with respect to primer design/selection to target specific nucleic acid sequences. As such, there is a need for a broad-range detection method for both anticipated viruses, as well as unanticipated viruses.

A study by the National Institutes of Health (NIH) in 2014 compared the sensitivity of in vivo assays and in vitro cell culture tests using a panel of 16 viruses and found the in vitro tests to be more sensitive for detecting most of the tested viruses^9^. The results from the NIH study support the use of tests for broader detection of adventitious viruses, particularly where no suitable animal models or appropriate culture methods for detection exist. The World Health Organization (WHO; WHO technical report series 978 [Annex 3])^10^ and the European Pharmacopoeia (legally binding standards and quality specifications; Ph. Eur. 2.6.16, Ph. Eur. 5.2.14, Ph. Eur. 5.2.3.)^10–13^ recommend or require that prior to the implementation of new alternative methods to detect adventitious agents, the specificity and sensitivity of the new and existing methods must be compared; and whether the new method has at least the same sensitivity as in vivo methods should be determined (Supplementary Table 1).

High-throughput sequencing (HTS) is a non-specific technique with the potential to detect both known and unknown adventitious agents including viruses^7,14–17^. High-throughput molecular biology methods (HTS combined with a pan-viral microarray) had succeeded in detecting the contamination of Rotarix vaccine by a porcine circovirus^18^. HTS may also be more sensitive than quantitative PCR (qPCR)^19,20^; however the method may be overly sensitive to the detection of background and cross-contaminating viral nucleic acids originating from the laboratory environment or from other sources^21^. Viral genome size can also influence the sensitivity of HTS^22,23^ as sensitivity is expected to be proportional to the mass ratio of nucleic acids in a given matrix. Although HTS results for adventitious virus testing may differ between laboratories^24^, the development of well-characterized model virus stocks would support standardization and validation of the different HTS platforms^22^. Currently there are no established viral reference standards with corresponding in vivo data, for assessing new techniques for the detection of adventitious viruses.

The panel of 16 virus stocks from the NIH is instrumental in our efforts to develop, qualify and validate the HTS adventitious virus test, by providing an important baseline against which new techniques for the detection of adventitious viruses can be compared^9^. Here, we describe the development of a general-purpose HTS-based test for the detection of adventitious viruses and its performance using virus stocks equivalent to the 16 NIH model viruses, plus another six viruses of interest. Detection of these viruses was assessed in a live Yellow Fever virus vaccine crude harvest matrix and a Vero cell substrate matrix to define the sensitivity (limit of detection [LOD]) and to demonstrate the specificity of the HTS test for adventitious virus testing.

## Results

### Identification of approximate LOD and viral spike detection in a viral vaccine crude harvest nucleic acid extraction

Nucleic acid recovery for both the Yellow Fever Virus vaccine crude harvest and the Vero cell substrate matrix are shown in Supplementary Note 1.

The number of sequence reads detected for each of the spiked viruses is listed in Table 1. All 22 model adventitious viruses were detected in at least one of the spike levels assessed. LOD was determined to be approximately 10^3^ to 10^4^ genome copies per mL of Yellow Fever Virus vaccine crude harvest. Specificity was assessed for all 22 viruses by determining whether the bioinformatics tool PhyloID was able to unequivocally detect each of the 22 spiked-in viruses and that none of these viruses was detected in the negative control (unspiked Yellow Fever virus vaccine crude harvest). Specificity was verified for all 22 viruses at the species level while specificity was verified for 20 of the 22 viruses at the strain level. For mammalian reovirus type 3 and coxsackievirus B3, the reads from the virus used for the spike were spread over two (for coxsackievirus B3) or three (for mammalian reovirus type 3) reference sequences of the correct viral species.

**Table 1:** Number of sequence reads detected for each of the spiked viruses at the different spiking levels in the viral vaccine crude harvest.

Virus	Spiking level (GC mL^−1^)					
	10^5^	10^4^	10^3^	10^3^	10^2^	10^2^
Total raw reads	291,341,932	371,184,528	365,131,932	333,373,964	437,070,286	332,181,730
NIH						
Adenoviridae						
Adenovirus 5	14,382	2284	264	193	34	34
Adenovirus 41	1154	164	12	16	11	6
Flaviviridae						
Bovine Viral Diarrhea Virus	95,666	6306	776	926	68	57
Herpesviridae						
Herpes Simplex Virus Type 1	1366	200	40	23	6	2
Simian Cytomegalovirus	49,266	9386	1170	676	116	87
Orthomyxoviridae						
Influenza A Virus	16,553	1266	83	166	8	3
Paramyxoviridae						
Bovine Parainfluenza Virus Type 3	48,578	3548	321	410	24	32
Measles Morbillivirus	12,571	922	87	105	0	10
Mumps Virus	9041	524	86	46	10	2
Picornaviridae						
Coxsackievirus A16	351	27	1	0	3*	0
Coxsackievirus B3	2036	157	10	18	9	8
Echovirus 11	2539	140	4	14	5*	1
Rhinovirus 2	1259	62	6	20	0	4
Polyomaviridae						
Simian Virus 40	528	88	47	15	10	3*
Rhabdoviridae						
Vesicular Stomatitis Virus	13,120	1071	102	157	27	12
Togaviridae						
Rubella Virus	4610	391	76	51	4	2
SP						
Bornaviridae						
Human Borna Disease Virus	341	6	1	0	0	0
Coronaviridae						
Bovine Coronavirus	7447	473	48	46	5*	2
Herpesviridae						
Human Cytomegalovirus	162,008	28,013	2733	2657	305	252
Parvoviridae						
Minute Virus of Mice	319	48	12	10	0	0
Porcine Parvovirus	146,720	20,668	2412	2609	397	238
Reoviridae						
Reovirus Type 3	11	0	0	0	0	0

Replicate spikes were made at 10^3^ and 10^2^ GC/mL as it was hypothesized that the LOD may be near this spike level.

*GC* genome copy, *NIH* National Institutes of Health, *SP* Sanofi Pasteur.

*3 or more reads belonging to one contig that is less than 201 bp (does not meet criteria for positive result).

### Assessment of LOD

Using a range of viral spikes from 10^5^ to 10^3^ genome copies per mL, most viruses were consistently detected at least at 10^4^ genome copies per mL in viral vaccine crude harvest (Table 2).

**Table 2:** Repeatability of the number of reads detected by the HTS test across 3 replicate datasets in the viral vaccine crude harvest.

Spiked virus	10^4^ GC mL^−1^ replicate 1	10^4^ GC mL^−1^ replicate 2	10^4^ GC mL^−1^ replicate 3	Mean number of reads at 10^4^ genome copies per mL	Standard deviation	% Coefficient of variation
NIH						
Adenoviridae						
Adenovirus 5	2284	1792	2570	2215	394	18
Adenovirus 41	164	120	176	153	29	19
Flaviviridae						
Bovine Viral Diarrhea Virus	6306	18,680	21,484	15,490	8076	52
Herpesviridae						
Herpes Simplex Virus Type 1	200	255	309	293	33	11
Simian Cytomegalovirus	9386	3,760	5,080	6,075	2942	48
Orthomyxoviridae						
Influenza A Virus	1266	21	33	5,408	3953	73
Paramyxoviridae						
Bovine Parainfluenza Virus Type 3	3548	8760	10,795	7701	3738	49
Measles Morbillivirus	922	1950	1778	1550	551	36
Mumps Virus	524	3602	5741	3289	2623	80
Picornaviridae						
Coxsackievirus A16	27	112	53	64	44	69
Coxsackievirus B3	157	969	986	704	474	67
Echovirus 11	140	847	1,854	947	861	91
Rhinovirus 2	62	238	197	166	92	55
Polyomaviridae						
Simian Virus 40	88	62	75	75	13	17
Rhabdoviridae						
Vesicular Stomatitis Virus	1071	9257	13,396	7908	6272	79
Togaviridae						
Rubella Virus	391	560	796	582	203	35
SP						
Bornaviridae						
Human Borna Disease Virus	6	403	447	285	243	85
Coronaviridae						
Bovine Coronavirus	473	1369	1813	1218	683	56
Herpesviridae						
Human Cytomegalovirus	28,013	139,737	66,743	162,203	108,453	67
Parvoviridae						
Minute Virus of Mice	104	130	1813	94	42	45
Porcine Parvovirus	20,668	54,941	15,775	30,461	21,341	70
Reoviridae						
Reovirus Type 3	0	120	49	56	60	107

*NIH* National Institutes of Health, *SP* Sanofi Pasteur.

### Reproducibility of the number of reads detected by the HTS test across three replicate datasets in the viral vaccine crude harvest

Reproducibility of the test was evaluated using three independent replicates (over two spiking studies) at a spiking level of 10^4^ genome copies per mL. The mean number of reads at 10^4^ genome copies per mL is shown in Table 2; the coefficient of variation ranged from 11 to 107%.

### Identification of approximate LOD and viral spike behavior in the cell substrate matrix

The number of sequence reads detected for each of the spiked viruses is listed in Table 3. For the Vero cell substrate with a cell count of 1 × 10^6^ cells per mL, 19 of the 22 viruses were detected at least at 10^4^ genome copies per mL (0.01 genome copies per cell) in the cell substrate matrix, with potentially better sensitivity for some viruses. Influenza A virus, minute virus of mice and reovirus 3 were not detected at this highest spike level of 0.01 genome copies per cell.

**Table 3:** Number of sequence reads detected for each of the spiked viruses at the different spiking levels in the cell substrate matrix.

Virus	Genome copies per cell			
	0.01	0.01	0.001	0.001
NIH				
Adenoviridae				
Adenovirus 5	244	125	18	4
Adenovirus 41	21	8	0	0
Flaviviridae				
Bovine Viral Diarrhea Virus	1014	1096	11	12
Herpesviridae				
Herpes Simplex Virus Type 1	58	17	0	0
Simian Cytomegalovirus	449	386	2	32
Orthomyxoviridae				
Influenza A Virus	0*	0*	0*	0*
Paramyxoviridae				
Bovine Parainfluenza Virus Type 3	627	512	25	11
Measles Morbillivirus	43	45	0	0
Mumps Virus	181	253	2	4
Picornaviridae				
Coxsackievirus A16	10	9	0	0
Coxsackievirus B3	55	106	0	0
Echovirus 11	110	86	0	0
Rhinovirus 2	6	20	0	0
Polyomaviridae				
Simian Virus 40	48	29	1	0
Rhabdoviridae				
Vesicular Stomatitis Virus	580	674	5	7
Togaviridae				
Rubella Virus	130	41	0	0
SP				
Bornaviridae				
Human Borna Disease Virus	14	10	0	9
Coronaviridae				
Bovine Coronavirus	35	41	0	2
Herpesviridae				
Human Cytomegalovirus	12,990	13,089	554	551
Parvoviridae				
Minute Virus of Mice	0*	14*	0*	0*
Porcine Parvovirus	5428	4932	327	214
Reoviridae				
Reovirus Type 3	0*	0*	0*	0*

Replicate spikes were made at each spiking level.

*GC* genome copy.

*indicates that the LOD for the specific virus was not identified through this study.

### LOD overview

Preliminary evaluation indicates that the adventitious virus detection test using HTS can potentially achieve a limit of detection that is at or below 0.01 genome copies per cell in a Vero cell substrate matrix (Table 3). 19 of the 22 viruses were detectable at 0.01 genome copies per cell and 14 of the viruses were detected at 0.001 genome copies per cell in a Vero cell substrate matrix (Table 3).

**Fig. 1:**
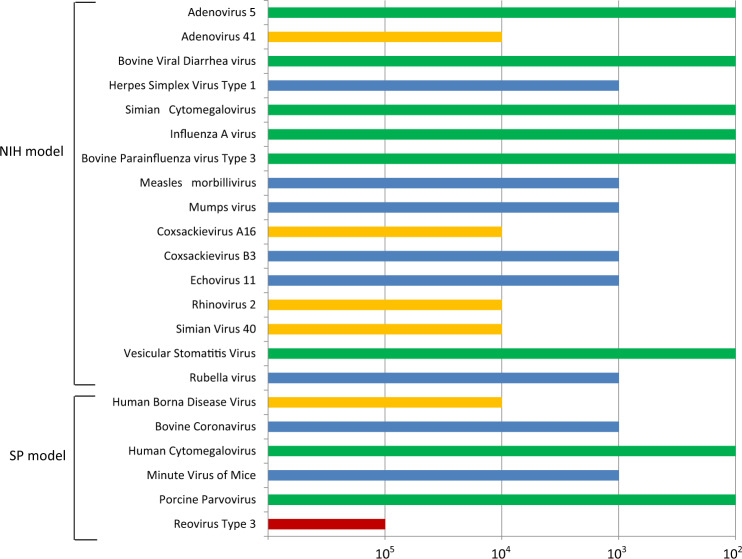
Limits of Detection for HTS assay for model adventitious viral agents in a Yellow Fever Vaccine viral crude harvests. NIH National Institutes of Health, SP Sanofi Pasteur. Graph shows limits of detection (LOD) in terms of genome copy number, with shorter bars indicating lower sensitivity (higher LOD). Colours denote the different LOD values, as follows: red, 10^5^; yellow, 10^4^; blue, 10^3^; and green, 10^2^.

In the Yellow Fever Virus vaccine crude harvest, HTS can achieve a LOD that is at or below 10^4^ genome copies per mL. Of the viruses assessed, 21 were detectable at 10^4^ genome copies per mL, with 16 of these at 10^3^ genome copies per mL in a matrix containing 10^9^ viral vaccine genome copies per mL (Fig. 1).

### Comparison to NIH in vivo testing results

In the previous NIH study, 11 of the 16 virus stocks were assessed for their detection in the in vivo test^9^. While the in vivo assays detect infectious virus, with levels reported as titers, the HTS method measures nucleic acids. We converted the sensitivity reported in the NIH study into genome copies using the infectious titer to genome copy ratio determined from the vial of original NIH viral stock. Nine of the 11 viruses previously assessed in vivo were detected with better sensitivity by our HTS method in the viral vaccine crude harvest matrix than reported in vivo in the NIH study; the remaining five viruses were not assessed in vivo within the NIH study (Table 4). Influenza A virus and VSV were detected with better sensitivity in vivo in the NIH study than using HTS in this study; these viruses were also detected at 10^4^-fold and 10^5^-fold lower genomic copy amounts in vivo compared to in vitro, respectively, as the animal model allows an efficient amplification of these replicating viral contaminants.

**Table 4:** Comparison of the sensitivity in a viral vaccine crude harvest versus sensitivity of the NIH in vivo tests.

NIH virus	In vivo LOD (TCID_50_, as described previously)^9^	Sensitivity of the in vivo assay (GC, determined by Clean Cell)	Sensitivity of the HTS assay (GC)
Adenoviridae			
Adenovirus 5	NT	NT	90
Adenovirus 41	NT	NT	9000
Flaviviridae			
Bovine Viral Diarrhea Virus	Not detected	≥3.39 × 10^8^	90
Herpesviridae			
Herpes Simplex Virus Type 1	100	3.4 × 10^4^	900
Simian Cytomegalovirus	NT	NT	90
Orthomyxoviridae			
Influenza A Virus	10^−4^	5.46 × 10^−4^*	90*
Paramyxoviridae			
Bovine Parainfluenza Virus Type 3	Not detected	≥8.67 × 10^7^	90
Mumps Virus	Undiluted	1.12 × 10^9^	900
Measles Morbillivirus	Not detected	≥4.00 × 10^8^	900
Picornaviridae			
Coxsackievirus A16	10^5^	3.03 × 10^8^	9000
Coxsackievirus B3	1	3.0 × 10^4^	900
Echovirus 11	Not detected	≥6.29 × 10^9^	900
Rhinovirus 2	NT	NT	9000
Polyomaviridae			
Simian Virus 40	NT	NT	9000
Rhabdoviridae			
Vesicular Stomatitis Virus	10^−4^	1.28 × 10^−4^*	90*
Togaviridae			
Rubella Virus	Not detected	≥2.45 × 10^7^	900

HTS assay is corrected for the volume used for extraction (900 μL).

*GC* genome copy, *HTS* high-throughput sequencing, *ND* Not detected, *NIH* National Institutes of Health, *NT*Not tested.

*viruses with lower sensitivity using the HTS test compared to the in vivo assay.

## Discussion

Various reviews of HTS for adventitious agent detection have shown that there is a need for unbiased extraction methods, relevant controls, the use of spike recovery experiments, and quality control measures during library preparation^7,14–17^. Our HTS method is a general-purpose non-specific method that can be used to detect both known and unexpected viruses in multiple types of samples (viral seeds/crude harvest, cell banks, and cell supernatants). The LOD for a panel of 22 model adventitious viruses was assessed in a viral vaccine crude harvest matrix and a cell substrate matrix. Based on the results, the adventitious virus test using HTS technology is capable of detecting adventitious viruses at least at 10^4^ genome copies per mL in the viral vaccine crude harvest matrix. Detection in the cell substrate matrix was at least at 0.01 viral genome copies per cell (10^4^ genome copies per mL) for 19 of the 22 viruses. In a similar study evaluating the performance of HTS for the detection of adventitious viruses by spiking model viruses into a cellular matrix containing whole cells, all of the model viruses used (human respiratory syncytial virus [RSV], Epstein-Barr virus [EBV], feline leukemia virus and human reovirus) were detected when spiked at 3 genome copies per cell; only EBV and RSV were detected at 0.1 genome copies per cell^22^. This study showed that HTS can detect almost all viral contaminants evidenced by the large panel that was investigated, i.e., 22 different viruses that were enveloped/non-enveloped, single-stranded/double-stranded, RNA, DNA and of varying genome sizes. The use of the method for different sample types was also demonstrated, i.e., the nucleic acid recovery from the viral vaccine crude harvest matrix was higher than the cell substrate matrix, however a high proportion of contaminants was detected for both samples. The difference in detection levels observed for some of the viruses is likely attributed to a higher amount of background host nucleic acids from the cell substrate. Compared to the vaccine viral harvest, where even if the cells are lysed during manufacturing, the host cell nucleic acids are limited within the viral harvest, which is very different when testing the cell substrate matrix. These cells are lysed during sample preparation, releasing their nucleic acids, and will therefore make up a higher proportion of the sequence data leading to a poorer signal (viral spike) to noise ratio and a decrease in the number of reads detected for the viral spikes. Overall, the successful detection of the spiked-in viruses in these complex backgrounds demonstrates the applicability of HTS for adventitious virus detection.

Methods for the detection of adventitious agents continue to evolve and improve. The use of viral stocks equivalent to the well-characterized viral stocks from the NIH-supported studies allows us to compare our HTS performance against compendial in vivo testing, as proposed in the European Pharmacopoeia Chapter 5.2.14^11^. In addition, such well-characterized materials can be used as a reference to help ensure satisfactory test performance in many matrices and thus, enable more meaningful comparisons between laboratories^24^. Importantly, the use of HTS is in-line with the Directive 2010/63/EU of the European Parliament and of the Council of 22 September 2010 with the aim, and the need, to reduce the number of animals used for scientific purposes^25^.

The approach to this study is based on European Pharmacopeia chapter 5.2.14 (Supplementary Table 1) and uses 16 well-characterized model viruses that are representative of potential contaminants in vaccine manufacturing to demonstrate that HTS sensitivity is at least equivalent to the sensitivity of the in vivo test methods. In this study, we produced 16 viruses that were equivalent to the viruses assessed in the NIH study. In vivo test results were available for 11 of the viruses in the NIH study and 9 of our 11 viruses were detected with better sensitivity by HTS in the viral vaccine crude harvest compared with the NIH in vivo data. Influenza A virus and VSV were the two viruses detected with lower sensitivity by HTS (also detected with lower sensitivity in vitro) which most likely suggests that the animal models used provide a good environment for the replication of those viruses, while HTS does not involve any viral replication. Two viruses resolved to the species level, but not to the strain level (mammalian reovirus type 3 and coxsackievirus B3) when measured by the in-house Sanofi Pasteur tool PhyloID^26^. However, resolution at the strain level can be more difficult when counts are low and when strains are similar in their sequences, as the reads may get assigned to any of the reference genomes within that species. We are, however, working on improving the strain resolution of PhyloID.

A limitation of a HTS adventitious virus detection method is that the test only detects viral nucleic acid and further investigation is necessary to determine if the signal is from infectious particles. In some cases, for example gamma-irradiated raw materials, or inactivated samples, the presence of viral nucleic acids is not necessarily a concern. In addition, HTS may be prone to detection of background and cross-contaminating viral nucleic acids originating from the laboratory environment or from other sources due to its breadth of detection, as well as its sensitivity^21^. A thorough understanding of the testing environment, proper test controls and knowledge of the history of the test sample will help to determine if laboratory follow-up is necessary.

Possible laboratory follow-ups include confirmation of intact full-length viral genomes^27,28^, expression analysis^29,30^ and infectivity assays^4,31^. It is also essential to validate the HTS test under Good Manufacturing Practice in order to apply the method to new vaccine products as release specification tests, as a substitution of in vivo tests to detect adventitious virus and to potentially replace/substitute specific tests such as PCRs used in routine testing. HTS would need to be compared with qPCR if it is to become routinely used. The sensitivity of HTS is influenced by genomic size^22,23^, as well as genomic structure, and for RNA viruses, the relative efficiencies in reverse transcription and cDNA synthesis^22^. Nevertheless, HTS is virus-sequence independent and can detect all types of virus genomes, including single-stranded/ double-stranded RNA and DNA.

The HTS method for adventitious virus detection is a highly sensitive, unbiased non-specific method, which has a large breadth of detection compared to other tests, a favorable comparison to in vivo data and may obviate the need to perform in vivo testing for adventitious viruses.

## Methods

**Fig. 2:**
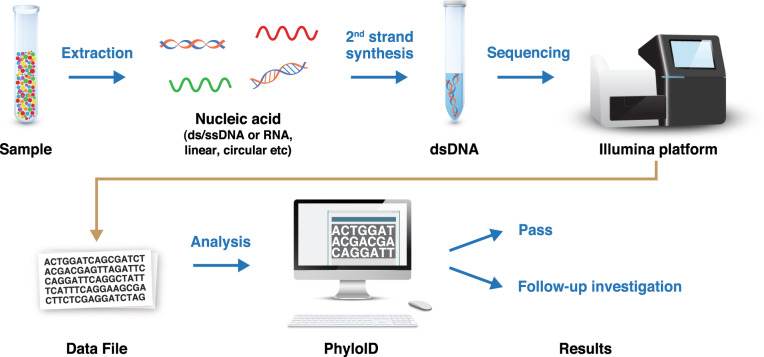
Overview of HTS for adventitious virus detection. Extraction of the sample in order to recover all types of nucleic acid, followed by second-strand synthesis and sequencing library preparation. Data analysis was carried out by PhyloID (a Sanofi Pasteur developed analysis pipeline). ds double-stranded, ss single-stranded.

A schematic overview of the HTS adventitious virus detection procedure is provided in Fig. 2.

### Preparation of virus panel

Two vials of each of the 16 virus stocks were received from the NIH; the viruses are described in Supplementary Table 2. The genome copy (GC) of each stock was determined by qPCR by Clean Cells (https://clean-cells.com/), a contract organization for Sanofi Pasteur, using plasmids containing synthetic nucleic acids of the targeted sequence to establish a standard curve; the resultant GC was used in addition to the infectious titer to convert the in vivo and in vitro LOD values established in the previous NIH study^9^ (expressed in infectious units) into equivalent genome copy numbers (the unit used for HTS detection) (Supplementary Table 3). Using these initial vials, larger viral stocks of similar characteristics were produced using protocols provided by the NIH for virus propagation and titration assays; the same sourced cells, culture medium, multiplicity of infection, infection duration, and harvest conditions were used. The production of viral stocks was conducted by Clean Cells. Six additional virus stocks of interest to Sanofi Pasteur that were available internally or from Clean Cells with the necessary viral titer and equivalent genome copy were also analyzed (Human Cytomegalovirus, Reovirus Type 3, Minute Virus of Mice, Porcine Parvovirus, Bovine Coronavirus and Human Borna Disease Virus; Supplementary Table 4). A viral pool was created at 1 × 10^6^ genome copies of each virus per mL in Tris EDTA. The volume of each virus necessary for the creation of the viral pool is listed in Supplementary Table 5; Tris-EDTA buffer solution was added to the viral pool to bring the final volume to 1 mL.

### Spiking experiments

Spiking experiments were designed based upon previous internal and collaborative studies^22,24^. Samples were prepared in a serum-free live Yellow Fever virus vaccine crude harvest and serum-free Vero cell substrate matrix; the same starting materials and extraction method were used for all experiments for consistency. The live Yellow Fever virus vaccine crude harvest was selected to represent a viral matrix for which HTS testing will be used at Sanofi Pasteur. The vaccine virus was titered at approximately 10^9^ viral vaccine genome copies per mL and the NIH-equivalent model adventitious viruses were spiked in at 10^5^, 10^4^, 10^3^, and 10^2^ genome copies per mL. Two replicate experiments were performed for spiking at both 10^3^ and 10^2^ genome copies per mL as it was hypothesized that the LOD for the assay may be close to this spiking level. We used a Vero cell-substrate matrix as a representative cell bank, which is used frequently at Sanofi Pasteur in the manufacture of several viral vaccines. Samples of the Vero cell substrate matrix (10^6^ cells per mL) were spiked at levels of 10^4^ and 10^3^ genome copies per mL (0.01 and 0.001 virus genome copies per cell, respectively). Replicate spikes were made at each spike level.

### Nucleic acid extraction, cDNA synthesis and assessment of the sample

**Fig. 3:**
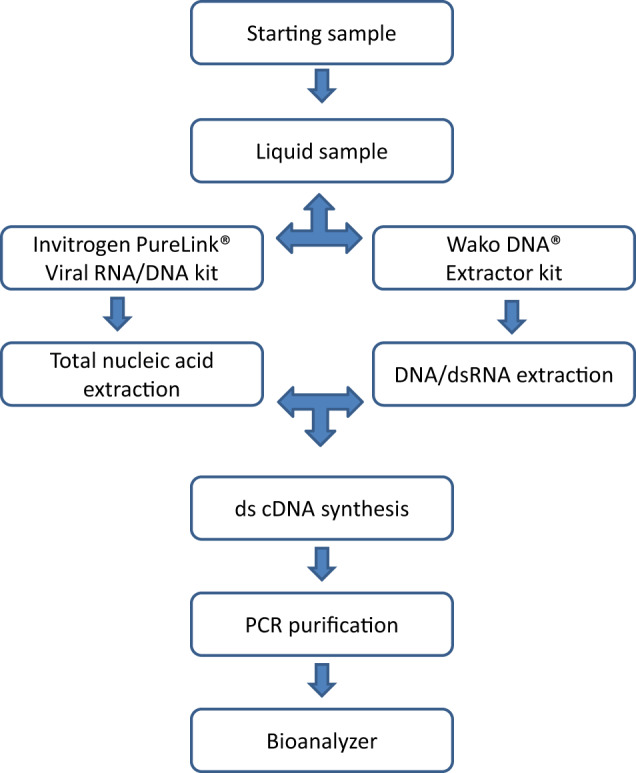
General overview of viral nucleic acid extraction protocol. cDNA complementary DNA, ds double-stranded, PCR polymerase chain reaction.

A schematic overview of the viral nucleic acid extraction protocol is presented in Fig. 3. Optimization of nucleic acid extraction has been described previously^32^ and was undertaken with the Invitrogen PureLink Viral RNA/DNA Mini kit (Life Technologies cat #12280050) and the Wako® DNA Extractor Kit (WAKO cat #295-50201). cDNA synthesis was undertaken with the SuperScript® Double-Stranded cDNA Synthesis kit (Invitrogen cat #11917-010), followed by nucleic acid purification using the QIAquick PCR Purification Kit (Qiagen cat #28104). The nucleic acid extractions were assessed using the Agilent 2100 Bioanalyzer system.

### Sequencing

Sequencing library preparation was performed using the Illumina Nextera XT DNA Library Prep kit. Sequencing was carried out on the Illumina HiSeq1500. Paired-end sequencing was carried out at 2 ×151 bp. Sequence data were converted from the Bcl to FASTQ formats using the Illumina Bcl2Fastq2 software.

### Data analysis

Data analysis was undertaken using PhyloID (a Sanofi Pasteur in-house bioinformatics tool developed for adventitious virus detection) and has been described previously^26^. Sequence data were trimmed, assembled into contigs and identified using a phylogenomic approach based on the profile of matches against reference data. Sensitivity was determined as the lowest concentration that a virus was conclusively positive (here, defined as >2 reads in >1 contig or in 1 contig >200 nt from the PhyloID analysis).

### Limit of detection (LOD)

The limit of detection of adventitious viruses using HTS was compared to the data obtained from the NIH study^9^. The LOD achieved by the in vivo test methods, as described in the NIH study, was converted to genome copies based on a determination of genome content of the NIH stocks by Clean Cells to facilitate the comparison with the HTS studies described here.

The genome copies at LOD for the in vivo test were calculated using the formula:

$$\frac{{{\mathrm{In}}\,{\mathrm{vivo}}\,{\mathrm{infectious}}\,{\mathrm{LOD}} \ast }}{{{\mathrm{Stock}}\,{\mathrm{infectious}}\,{\mathrm{iter}} \ast }} \times {\mathrm{Genome}}\,{\mathrm{copies}}\,{\mathrm{per}}\,{\mathrm{mL}}\,{\mathrm{of}}\,{\mathrm{stock}}\dagger = {\mathrm{Genome}}\,{\mathrm{copies}}\,{\mathrm{at}}\,{\mathrm{LOD}}$$

For example, for Herpes Simplex Virus Type 1:

$$\frac{{10^2{\mathrm{PFU}}}}{{9.5 \times 10^6{\mathrm{PFU}}\,{\mathrm{mL}}^{ - 1} \ast }} \times (3.23 \times 10^9\,{\mathrm{genome}}\,{\mathrm{copies}}\,{\mathrm{per}}\,{\mathrm{mL}}\dagger ) = 34,000\,{\mathrm{genome}}\,{\mathrm{copies}}$$

*All obtained from the NIH stock^9^, ^†^As determined by Clean Cells from the NIH stock.

(The limit of detection for viruses not detected in vivo is specified as greater than the genome copy number after conversion from the titer of the NIH stock virus [Supplementary Tables 2 and 3]).

## Supplementary information

Supplementary Information

## Data Availability

The data that support the findings of this study are available on request from the corresponding author (S.N.).
