# Supplementary material for: Sensitivity and breadth of detection of high-throughput sequencing for adventitious virus detection
Source: NPJ Vaccines. 2020 Jul 17;5:61. doi: 10.1038/s41541-020-0207-4 (PMC7368052; doi:10.1038/s41541-020-0207-4)
Supplement: Supplementary file 1 — Supplementary Information [file 41541_2020_207_MOESM1_ESM.pdf]

Supplementary information

Supplementary Note 1

Nucleic acid recovery

Viral vaccine crude harvest

The nucleic acids recovered were 60–137 pg μL^{-1} and the library concentration range was 2.8–4.5 ng mL^{-1}.

Cell substrate matrix

Nucleic acid recovery was 4.5–6.6 ng μL^{-1} and the Library concentration range was 2.4–3.0 ng μL^{-1}.

Supplementary Table 1: The World Health Organization and European pharmacopeia recommendations for new methods to detect adventitious agents

Reference	Recommendation
WHO TRS 978 Annex 3 - Cell substrates:[1]	"New and sensitive molecular methods with broad detection capabilities are being developed. These are not yet in routine use but, as they become widely available and validated, they will play an increasing role in the evaluation of cell substrates. The sensitivity of these methods, as well as their breadth of detection, should be considered when evaluating their applicability."
WHO TRS 978 Annex 5: Live Attenuated Yellow Fever:[1]	"New molecular methods with broad detection capabilities are being developed for the detection of adventitious agents. These methods include: degenerate NAT for whole virus families with analysis of the amplicons by hybridisation, sequencing or mass spectrometry; NAT with random primers followed by analysis of the amplicons on large oligonucleotide microarrays of conserved viral sequencing or digital subtraction of expressed sequences; and high-throughput sequencing. These methods may be used in the future to supplement existing methods, or as alternative methods to both *in vivo* and *in vitro* tests, after appropriate validation and approval by the NRA. "<br>same paragraph in WHO TRS 979 Annex 2 (Live attenuated Dengue Vaccine)
Ph. Eur 5.2.3: "Cell Substrates for the production of vaccines for human use", version 9:0 and updated version 9.3 in July 2017, revision[2]	"New molecular methods with broad detection capabilities are being developed for the detection of adventitious agents. These methods include: degenerate NAT for whole virus families with analysis of the amplicons by hybridisation, sequencing or mass spectrometry; NAT with random primers followed by analysis of the amplicons on large oligonucleotide microarrays of conserved viral sequencing or digital subtraction of expressed sequences; and high-throughput sequencing. These methods may be used in the future to supplement existing methods, or as alternative methods to both *in vivo* and *in vitro* tests, after appropriate validation and approval by the NRA."
Ph. Eur. 5.2.14: "Substitution of in vivo method(s) by in vitro method(s) for the quality control of vaccines", version 9.3 published in July 2017, creation[3]	<u>Detection of viral extraneous agents by novel molecular methods:</u> Detection of viral extraneous agents in cell banks, seed lots and cell culture harvests is currently conducted using a panel of *in vivo* and *in vitro* methods at different stages of the manufacturing process. Novel, sensitive molecular techniques with broad detection capabilities are available, including deep sequencing or high throughput sequencing methods, degenerate PCR for whole virus families or random-priming methods (associated or not with sequencing), hybridisation to oligonucleotide arrays and mass spectrometry. The use of these new broad molecular methods has highlighted the gaps with the existing testing strategy by identifying previously undetected viral contaminants in final product, the cell banks from which it was produced and intermediate manufacturing stages. These new broad molecular methods (e.g. deep sequencing or high throughput sequencing) detect genomes while the existing *in vivo*

	methods are based on observations of the effects viruses have on experimental animals. The implementation of such new broad molecular methods as substitutes for *in vivo* methods requires a comparison of the specificity (breadth of detection) and the sensitivity of the new and existing methods. For this purpose, an appropriate panel of representative well-characterised model viruses should be used to assess the ability of the new method to detect viruses that are (or are not) detected by the *in vivo* methods and, to determine if the sensitivity is at least equivalent to the sensitivity of the *in vivo* methods. This last element is particularly complex since these new molecular methods do not detect the same characteristic of the viral contaminant (genome for molecular methods versus infectious virus for *in vivo* methods) and also the fact that no or limited validation data exist for the *in vivo* methods. It should be also emphasised that the outcome of the broad molecular methods is not the final result since the detection of a genome or fragments of a genome does not necessarily indicate the presence of an infectious virus.”
Ph . Eur . 2.6.16: ”Tests for extraneous agents in viral vaccines for human use”, version 9.3 published in July 2017, revision. Corrected version in Supplement 9.4[4]	“New molecular methods with broad detection capabilities are being developed for the detection of adventitious agents. These methods include: degenerate NAT for whole virus families with analysis of the amplicons by hybridisation, sequencing or mass spectrometry; NAT with random primers followed by analysis of the amplicons on large oligonucleotide microarrays of conserved viral sequencing or digital subtraction of expressed sequences; and high-throughput sequencing. These methods may be used in the future to supplement existing methods, or as alternative methods to both *in vivo* and *in vitro* tests, after appropriate validation and approval by the NRA.”

3 Ph. Eur., The European Pharmacopoeia general chapters; WHO, the World Health Organization

Supplementary Table 2: NIH Model Viruses

Viral Family and virus	Strain	Enveloped	Viral genome	Genome Size (Kb)	Production Cell Line
Adenoviridae					
Adenovirus 5	Adenoid 75	No	dsDNA	36	A549
Adenovirus 41	N/A	No	dsDNA	34	HEK293
Flaviviridae					
Bovine Viral Diarrhea Virus	NY-1	Yes	ssRNA (+ve)	12.4	BT
Herpesviridae					
Herpes Simplex Virus Type 1	MacIntyre	Yes	dsDNA	150	Vero
Simian Cytomegalovirus	CS6	Yes	dsDNA	221	MRC-5
Orthomyxoviridae					
Influenza A Virus	A/PR/8/34 (H1N1)	Yes	ssRNA (-ve)	12.5	MDCK
Paramyxoviridae					
Bovine Parainfluenza Virus Type 3	N/A	Yes	ssRNA (-ve)	15.5	Vero
Measles Morbillivirus	Edmonston	Yes	ssRNA (-ve)	15.9	Vero
Mumps Virus	Enders	Yes	ssRNA (-ve)	15.4	Vero
Picornaviridae					
Coxsackie virus A16	N/A	No	ssRNA (+ve)	7.4	Vero
Coxsackie virus B3	N/A	No	ssRNA (+ve)	7.4	LLC-MK2
Echovirus 11	Gregory	No	ssRNA (+ve)	7.4	LLC-MK2
Rhinovirus 2	HGP	No	ssRNA (+ve)	7.1	HeLa
Polyomaviridae					
Simian Virus 40	Pa-57	No	dsDNA	5.2	Vero
Rhabdoviridae					
Vesicular Stomatitis Virus	Indiana	Yes	ssRNA(-ve)	11.2	Vero
Togaviridae					
Rubella Virus	M-33	Yes	ssRNA (+ve)	9.7	BSC-1

ds, double stranded; NIH, National Institutes of Health; ss, single stranded.

7 **Supplementary Table 3: Genome copy number as determined by Clean Cells[†] for the NIH Stock**

	Lot #	NIH Stock for Seed			Clean Cells Production	
		**NIH Titer $TCID_{50}$ mL^{-1}***	**Clean Cells Titer $TCID_{50}$ mL^{-1}***	**Genome copies per mL**	**Infectious Titer $TCID_{50}$ mL^{-1}***	**Genome copies per mL**
Adenoviridae						
Adenovirus 5	SP-NIH-AD5-01	3.0×10^{8}<br>(Day 7)	4.27×10^{7}<br>(Day 7)<br>2.95×10^{9}<br>(Day 12)	2.56×10^{10}	2.95×10^{7}<br>(Day 12)	4.83×10^{8}
Adenovirus 41	SP-NIH-AD41-01	2.0×10^{5}	9.33×10^{5}	1.43×10^{11}	1.74×10^{5}	1.41×10^{10}
Flaviviridae						
Bovine Viral Diarrhea Virus	SP-NIH-BVDV-01	3.0×10^{7}<br>$FAID_{50}$ mL^{-1}	4.68×10^{7}	3.39×10^{8}	7.76×10^{7}	3.69×10^{8}
Herpesviridae						
Herpes Simplex Virus Type 1	SP-NIH-HSV1-01	9.5×10^{6}<br>PFU mL^{-1}	1.82×10^{6}<br>PFU mL^{-1}	3.23×10^{9}	5.50×10^{6}<br>PFU mL^{-1}	3.88×10^{8}
Simian Cytomegalovirus	SP-NIH-SCMV-05	3.0×10^{4}	1.35×10^{3}	3.28×10^{6}	9.33×10^{5}	5.44×10^{7}
Orthomyxoviridae						
Influenza A Virus	SP-NIH-Flu-01	6.3×10^{7}	2.95×10^{7}	3.44×10^{8}	2.57×10^{4}	6.21×10^{6}
Paramyxoviridae						
Bovine Parainfluenza Virus Type 3	SP-NIH-BPI3-01	6.3×10^{7}	2.00×10^{7}	8.67×10^{7}	8.13×10^{7}	1.57×10^{8}
Measles Morbillivirus	SP-NIH-Mea-01	9.3×10^{5}	2.95×10^{6}	4.00×10^{8}	9.33×10^{6}	3.25×10^{8}
Mumps Virus	SP-NIH-Mum-01	1.3×10^{6}	2.95×10^{6}	1.12×10^{9}	4.90×10^{6}	3.05×10^{9}
Picornaviridae						
Coxsackievirus, A16	SP-NIH-CAV-02	6.3×10^{6}	6.31×10^{5}	1.91×10^{10}	1.35×10^{3}	2.65×10^{8}
Coxsackievirus B3	SP-NIH-CBV-01	3.0×10^{7}	4.27×10^{7}	8.99×10^{9}	4.27×10^{6}	3.96×10^{8}
Echovirus 11	SP-NIH-Echo-01	2.0×10^{7}	9.33×10^{7}	6.92×10^{9}	1.20×10^{8}	3.56×10^{9}
Rhinovirus 2	SP-NIH-Rhin-03	1.3×10^{3}	2.95×10^{5}	1.09×10^{10}	2.24×10^{6}	4.76×10^{9}
Polyomaviridae						
Simian Virus 40	SP-NIH-SV40-01	7.1×10^{7}<br>PFU mL^{-1}	6.92×10^{7}<br>PFU mL^{-1}	4.14×10^{11}	1.78×10^{8}<br>PFU mL^{-1}	3.76×10^{11}
Rhabdoviridae						
Vesicular Stomatitis Virus	SP-NIH-VSV-02	1.0×10^{9}<br>PFU mL^{-1}	3.24×10^{7}	1.28×10^{9}	9.12×10^{8}	3.14×10^{9}
Togaviridae						
Rubella Virus	SP-NIH-Rub-02	1.3×10^{4}	4.27×10^{4}	2.45×10^{7}	8.71×10^{4}	1.72×10^{7}

*All titers are expressed as $TCID_{50}$ mL^{-1} unless otherwise indicated

[†]Clean Cells (https://clean-cells.com/) is a contract organization for Sanofi Pasteur

FAID, fluorescent antibody infectious dose; NIH, National Institutes of Health; PFU, plaque forming units; TCID, tissue culture infective dose

Supplementary Table 4: Additional Sanofi Pasteur viruses to complement the NIH model viruses

Virus	Strain	Enveloped	Viral genome	Genome size (Kb)	Infectious titer (log $CCID_{50}$ mL^{-1})	Genome copies per mL
Bornaviridae						
Human Borna Disease Virus	He/80/FR (BNV)	Yes	ssRNA	8.9	-	8.40×10^{8}
Coronaviridae						
Bovine Coronavirus	C-197	Yes	ssRNA	31	-	8.10×10^{6}
Herpesviridae						
Human Cytomegalovirus	Towne	Yes	dsDNA	236	7.85	7.07×10^{8}
Parvoviridae						
Minute Virus of Mice	Prototype	No	ssDNA	5.1	-	1.59×10^{12}
Porcine Parvovirus	NADL-2	No	ssDNA	5.1	-	1.24×10^{7}
Reoviridae						
Reovirus Type 3	Dearing	No	dsRNA	23.5	-	4.89×10^{9}

$CCID_{50}$, cell culture infectious dose 50% i.e. the quantity of infectious virus that when inoculated into susceptible cell cultures will infect 50% of the individual cultures

Viruses were obtained from the National Institutes of Health (NIH) National Institute of Allergy and Infectious Diseases (NIAID) and were made available by Rebecca Sheets (based on the Gombold et al. publication[5]).

NIH, National Institutes of Health.

Supplementary Table 5: Volume of each virus used to create the viral pool

Virus	Genome copies per mL	Amount required for 10^6 genome copies per mL	Additional dilutions of the viral stock	Final volume in spike (µL)
NIH model				
Adenoviridae				
Adenovirus 5	4.79×10^5	2.09	NA	2.09
Adenovirus 41	1.41×10^7	0.0709	1/100****	7.09
Flaviviridae				
Bovine Viral Diarrhea Virus	3.72×10^5	2.69	NA	2.69
Herpesviridae				
Herpes Simplex Virus Type 1	6.14×10^5	1.63	NA	1.63
Simian Cytomegalovirus	5.44×10^4	18.38	NA	18.38
Orthomyxoviridae				
Influenza A virus	2.24×10^3	446.43	NA	446
Paramyxoviridae				
Mumps Virus	3.02×10^6	0.331	1/10	3.31
Bovine Parainfluenza Virus Type 3	1.58×10^5	6.33	NA	6.33
Measles Morbillivirus	3.24×10^5	3.09	NA	3.09
Picornaviridae				
Coxsackie virus A16	2.65×10^5	3.77	NA	3.77
Coxsackie virus B3	3.96×10^5	2.53	NA	2.53
Echovirus 11	3.55×10^6	0.282	1/10	2.82
Rhinovirus 2	4.76×10^6	0.21	1/10	2.1
Polyomaviridae				
Simian Virus 40	3.80×10^8	0.00263	1/1000***	2.63
Rhabdoviridae				
Vesicular Stomatitis Virus	3.16×10^6	0.316	1/10	3.16
Togaviridae				
Rubella Virus	1.72×10^4	58.14	NA	58.1
SP model				
Herpesviridae				
Human Cytomegalovirus*	7.08×10^5	1.41	NA	1.41
Reoviridae				
Reovirus Type 3	4.89×10^6	0.204	1/10	2.04

Parvoviridae				
Minute Virus of Mice	1.59 x 10^{9}	0.000629	1/10 000**	6.29
Porcine Parvovirus	1.24 x 10^{4}	80.65	NA	80.6
Coronaviridae				
Bovine Coronavirus	8.10 x 10^{3}	123.46	NA	123.5
Bornaviridae				
Human Borna Disease Virus	8.40 x 10^{5}	1.19	NA	1.19

* For human cytomegalovirus, it was estimated that there may be on average 10 times as many genome copies as the CCID50 value of 7.85 log CCID50 mL^{-1}

** 10 µL of the viral stock was mixed with 990 µL of Tris EDTA to create a 1/100 dilution. 10 µL of the diluted virus was mixed with 990 µL of Tris EDTA to achieve the final 1/10 000 dilution

*** 1 µL of the viral stock was mixed with 9 µL of Tris EDTA to create a 1/10 dilution. 10 µL of the diluted virus was mixed with 990 µL of Tris EDTA to achieve the final 1/1000 dilution

**** 10 µL of the viral stock was mixed with 990 µL of Tris EDTA to create a 1/100 dilution.

GC, Genome copy; NA, not applicable; NIH, National Institutes of Health; SP, Sanofi Pasteur.

Supplementary references

1 WHO TRS 978 Annex. Available at: https://www.who.int/biologicals/vaccines/TRS_978_Annex_3.pdf?ua=1. Last accessed July 2019.

2 Ph. Eur. Chapter 5.2.3: “Cell Substrates for the production of vaccines for human use”, version 9:0 and updated version 9.3 in July 2017, revision.

3 Ph. Eur. Chapter 5.2.14: “Substitution of in vivo method(s) by in vitro method(s) for the quality control of vaccines”, version 9.3 published in July 2017, creation.

4 Ph. Eur. Chapter 2.6.16: “Tests for extraneous agents in viral vaccines for human use”, version 9.3 published in July 2017, revision. Corrected version in Supplement 9.4.

5 Gombold, J. *et al.* Systematic evaluation of in vitro and in vivo adventitious virus assays for the detection of viral contamination of cell banks and biological products. *Vaccine* **32**, 2916-2926, doi:10.1016/j.vaccine.2014.02.021 (2014).
